# Maintaining population health in low‐ and middle‐income countries during the COVID‐19 pandemic: Why we should be investing in Community Health Workers

**DOI:** 10.1111/tmi.13498

**Published:** 2020-10-12

**Authors:** Benjamin Palafox, Alicia Renedo, Gideon Lasco, Lia Palileo‐Villanueva, Dina Balabanova, Martin McKee

**Affiliations:** ^1^ Faculty of Public Health & Policy London School of Hygiene & Tropical Medicine London UK; ^2^ Department of Anthropology University of the Philippines Diliman Quezon City Philippines; ^3^ College of Medicine University of the Philippines Manila Manila Philippines; ^4^ Department of Global Health & Development London School of Hygiene & Tropical Medicine London UK

**Keywords:** community health workers, Covid‐19 responses, non‐communicable disease management, primary health care, low‐ and middle‐income countries

## Abstract

Community health workers in low‐ and middle‐income country primary health care systems are well suited to perform essential functions on the frontlines of Covid‐19 pandemic responses. However, clear and coordinated guidance, updated infection control training, and reliable access to personal protective equipment must be ensured in order to deploy them safely and effectively. With these additional responsibilities, community health workers must also be supported to ensure that hard‐fought gains in population health, including progress on non‐communicable diseases, are sustained throughout the pandemic.

The first wave of the COVID‐19 pandemic placed enormous pressures on health systems. The global news media has reported far too many examples of heroic health workers struggling with little support and inadequate resources against an invisible foe. Yet in many countries, these health workers were barely coping before the pandemic in systems that had failed to attract and retain skilled staff. The need for new solutions has never been greater. Writing about the United Kingdom, a country that is, in relative terms, well‐endowed with health workers, including many recruited from poorer countries, Haines *et al*. [1] have called for a national cadre of community health workers (CHWs) to support the UK’s ongoing COVID‐19 response.

CHWs have existed for many years in countries at all levels of economic development. The best‐known examples come from low‐ and middle‐income countries (LMICs), where large‐scale CHW programmes have been used as a cost‐effective means of undertaking tasks that would otherwise be done by the few doctors and nurses available, providing people‐centred basic health care to the underserved, while encouraging meaningful community participation in the health system [2, 3]. In all settings, their tasks typically include health promotion and disease prevention activities, collecting community health information, and the provision of basic treatment for common and easily managed conditions [4]. There is now extensive evidence from LMICs that CHWs, who typically commence work with little or no formal training, can effectively treat uncomplicated communicable diseases such as malaria, diarrhoea and pneumonia in the community [5, 6].

These experiences would suggest that CHWs are, indeed, well‐placed to play a role in national COVID‐19 responses. Roles might include regular monitoring of vulnerable people at home and, if individuals develop symptoms, conducting simple assessments, referring them for formal care as appropriate and collecting essential surveillance data [1]. Thus, there is a strong case for deploying CHWs in any health system under pressure, including the UK as suggested in *The Lancet* where 750 000 people volunteered to help the National Health Service [7]. However, the scope for adopting this policy and the resulting benefits are likely to be much greater in LMICs, particularly in those countries where large cadres of CHW already exist.

Our recent experience with the RESPOND project in the Philippines supports this view [8]. Filipino CHWs (called *Barangay Health Workers,* BHWs) have been deployed by the national government to community COVID‐19 emergency response teams [9]. Over the past four decades, BHWs have established themselves as integral members of the primary care workforce. Alongside their skills in communicating public health messages and providing a link between the population and the formal health system, they already know who is vulnerable and at risk from COVID‐19, including older people, those with pre‐existing conditions, and those without family support. As they are already relied upon for a wide range of health and social care support, CHWs are ideally placed to ensure households are aware of basic hygiene measures and can combat misinformation, identify and refer possible new cases, and monitor adverse effects of the disease or social distancing measures. Importantly, as lay representatives working within local health systems, CHWs act as natural conduits for community participation by gathering and feeding back the views and concerns of the most vulnerable and overlooked, so that COVID‐19 responses may be informed by community voices, something that has often been overlooked in many countries [10]. Notably, CHWs performed most, if not all, of these functions effectively during the most recent Ebola outbreak in West Africa and were instrumental in the success of national responses [11].

There are, however, several important considerations before CHWs in LMICs are deployed as part of the COVID‐19 response, as illustrated by Haines *et al*. [1] and by the Ebola outbreak responses in Liberia and Sierra Leone [11]. First, clear and coordinated guidance that defines and recognises the contribution of CHWs to the COVID‐19 response must be developed and endorsed across all levels of the health system. Also, many current CHWs are older women, placing them at greater risk of severe illness and death, as has been the case in health workforces around the world. Consequently, many existing CHWs may be unwilling or unable to assume such additional tasks, especially where they have caring responsibilities within their own families and social circles. To minimise the risk of infection to themselves and the community members they care for, CHWs must also receive updated infection control training and have reliable access to adequate personal protective equipment. These critical challenges have been noted in the Philippines [12], just as in some well‐resourced health systems [13], and have already contributed to the loss of at least 50 CHWs in Brazil [14].

Crucially, CHWs in LMICs must be supported to continue their normal duties to minimise the negative impact of the pandemic on the many population health gains they have worked hard to achieve. Beyond their historical contribution to maternal, neonatal and child health, the role of CHWs has gradually been expanding to include aspects of care for non‐communicable disease (NCD) in both higher‐ and lower‐income country settings [15, 16]. This is particularly important given that the negative health and economic impacts of the growing NCD burden fall disproportionately on the poor and vulnerable.

Our experience in the Philippines has illustrated the many ways that CHWs support people in low‐income communities to self‐manage existing long‐term conditions [17]. For example, CHWs help to ensure that individuals have sufficient medicine supplies, adhere to treatment regimes, and attend follow‐up appointments. They monitor blood pressure and glucose levels in homes and advise when people should seek more advanced care. This is of growing importance as, in many countries, we are already witnessing the consequences of individuals avoiding urgent care for non‐COVID illnesses, such as stroke and heart attack [18], with governments pivoting resources towards COVID care at the expense of other health services [19]. CHWs, in addition, can facilitate telemedicine and other innovations that have been spurred by the pandemic that have thus far only been tested in other settings and at small scale [20].

As it is now evident that the pandemic will continue for many more months, if not years, primary care systems in LMICs must adapt fully to this new reality. To assist this process, a variety of resources and operational policies have been developed on, for example, modifying routine practices to minimise unnecessary contact, and integrating new ones as part of the response [21, 22]. Where they already exist, CHWs operate on the frontline, especially in LMICs with vulnerable health systems, helping to keep not only the pandemic, but also its wider health consequences in check. Pandemic planning must consider the needs of CHWs to ensure that they are well‐equipped, trained and supported to perform what has become a vital role.
